# Albuminuria Is Associated with Hepatic Iron Load in Patients with Non-Alcoholic Fatty Liver Disease and Metabolic Syndrome

**DOI:** 10.3390/jcm10143187

**Published:** 2021-07-20

**Authors:** Manuela Abbate, Sofía Montemayor, Catalina M. Mascaró, Miguel Casares, Cristina Gómez, Lucía Ugarriza, Silvia Tejada, Itziar Abete, M. Ángeles Zulet, Antoni Sureda, J. Alfredo Martínez, Josep A. Tur

**Affiliations:** 1 Research Group in Community Nutrition and Oxidative Stress, University of the Balearic Islands-IUNICS, 07122 Palma de Mallorca, Spain; manuela.abbate@uib.es (M.A.); sofiamf16@gmail.com (S.M.); c.mascaro@uib.es (C.M.M.); lugarriza@ibsalut.caib.es (L.U.); silvia.tejada@uib.es (S.T.); antoni.sureda@uib.es (A.S.); 2 Health Research Institute of Balearic Islands (IdISBa), 07120 Palma de Mallorca, Spain; 3 Radiodiagnosis Service, Red Asistencial Juaneda, 07011 Palma de Mallorca, Spain; casaresmiguel@gmail.com; 4 Clinical Analysis Service, University Hospital Son Espases, 07120 Palma de Mallorca, Spain; cristina.gomez@ssib.es; 5 Camp Redó Primary Health Care Center, 07010 Palma de Mallorca, Spain; 6 CIBER Physiopathology of Obesity and Nutrition (CIBEROBN), Instituto de Salud Carlos III (ISCIII), 28029 Madrid, Spain; iabetego@unav.es (I.A.); mazulet@unav.es (M.Á.Z.); jalfredo.martinez@imdea.org (J.A.M.); 7 Center for Nutrition Research, Department of Nutrition, Food Sciences, and Physiology, University of Navarra, 31008 Pamplona, Spain; 8 Cardiometabolics Precision Nutrition Program, IMDEA Food, CEI UAM-CSIC, 28049 Madrid, Spain

**Keywords:** albuminuria, non-alcoholic fatty liver disease, hepatic iron load, fasting insulin, serum ferritin, platelet count, insulin resistance, metabolic syndrome

## Abstract

Background: Increased albuminuria is associated with increased serum ferritin, insulin resistance, and non-alcoholic fatty liver disease (NAFLD). Liver iron accumulation is also related to hyperferritinemia, insulin resistance, and NAFLD; however, there is no evidence on its relationship with albuminuria. Aims: To assess the relationship between hepatic iron load and urine albumin-to-creatinine ratio (UACR) in patients with metabolic syndrome (MetS) and NAFLD. Methods: In total, 75 MetS and NAFLD patients (aged 40–60 years, BMI 27–40 kg/m^2^) were selected from a cohort according to available data on hepatic iron load (HepFe) by magnetic resonance imaging (MRI). Subjects underwent anthropometric measurements, biochemistry testing, and liver MRI. Increased albuminuria was defined by UACR. Results: UACR correlated with NAFLD, HepFe, triglycerides, serum ferritin, fasting insulin, insulin resistance (calculated using the homeostatic model assessment for insulin resistance—HOMA-IR- formula), and platelets (*p* < 0.05). Multiple regression analysis adjusted for gender, age, eGFR, HbA1c, T2DM, and stages of NAFLD, found that HepFe (*p* = 0.02), serum ferritin (*p* = 0.04), fasting insulin (*p* = 0.049), and platelets (*p* = 0.009) were associated with UACR (R^2^ = 0.370; *p* = 0.007). UACR, liver fat accumulation, serum ferritin, and HOMA-IR increased across stages of HepFe (*p* < 0.05). Patients with severe NAFLD presented higher HepFe, fasting insulin, HOMA-IR, and systolic blood pressure as compared to patients in NAFLD stage 1 (*p* < 0.05). Conclusion: Hepatic iron load, serum ferritin, fasting insulin, and platelets were independently associated with albuminuria. In the context of MetS, increased stages of NAFLD presented higher levels of HepFe. Higher levels of HepFe were accompanied by increased serum ferritin, insulin resistance, and UACR. The association between iron accumulation, MetS, and NAFLD may represent a risk factor for the development of increased albuminuria.

## 1. Introduction

Non-alcoholic fatty liver disease (NAFLD), considered the hepatic expression of the metabolic syndrome, affects more than 25% of the Western population, and its number continues to increase [1]. A mild increase of liver iron accumulation and moderate hyperferritinemia in the context of the metabolic syndrome (MetS) have been detected in about half of patients with non-alcoholic fatty liver disease (NAFLD) [2,3]. Such condition has been named dysmetabolic iron overload syndrome (DIOS), and it is unrelated to hereditary hemochromatosis (HHC). DIOS may significantly influence the progression of NAFLD to more advanced liver injury and lead to an array of health consequences such as bone disease, diabetes, cardiovascular disease, hypothyroidism, or depression, amongst others [3].

Hyperferritinemia, besides being related to hepatic iron deposits, is also a determinant of NAFLD [4,5]. Moreover, it has been described as an early marker of insulin resistance [6,7], and is associated with increased albuminuria in patients with prediabetes and diabetes [8,9,10].

Albuminuria, defined as the ratio between urine albumin and urine creatinine (UACR), starting from the higher end of the normal range, is the renal manifestation of MetS and a recognized risk factor for cardiovascular events and progression to overt nephropathy [11,12,13,14,15,16].

Several studies have investigated the association between NAFLD and UACR and concluded that patients with NAFLD have a significantly increased risk of albuminuria, and that such risk may be mediated by insulin resistance [17]. Of note, the prevalence of NAFLD is higher in individuals with features of MetS such as obesity, diabetes, and dyslipidaemia [18,19]; and in turn, the presence of MetS increases the likelihood of developing more advanced forms of NAFLD [3,20,21].

Hyperferritinemia, insulin resistance, and NAFLD are all related to each other and significantly contribute to the risk of albuminuria. Liver iron accumulation is also related to hyperferritinemia, insulin resistance, and NAFLD; however, there is no evidence so far on whether it is also associated with albuminuria.

This study aimed to assess the relationship between hepatic iron load (HepFe) and urine albumin-to-creatinine ratio (UACR) in patients with metabolic syndrome (MetS) and NAFLD.

## 2. Methods

The baseline data used for the present secondary analysis belong to an ongoing multicenter randomized controlled trial, which compares two Mediterranean diets against a control diet on the progression of NAFLD in patients with MetS.

### 2.1. Subjects

**Figure 1:**
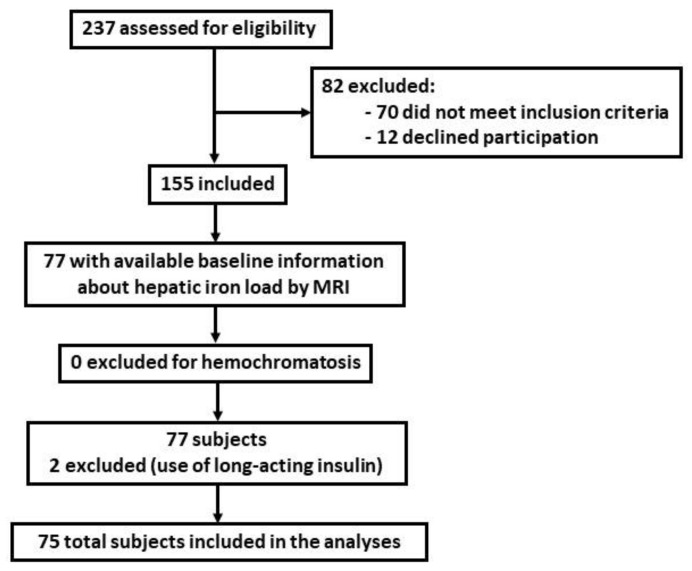
Study flow-chart.

The original ongoing study involves 155 participants between 40 and 60 years, with a diagnosis of NAFLD by magnetic resonance imaging (MRI) with a BMI of 27–40 kg/m^2^, and meeting at least three of the five criteria of MetS as described in the International Diabetes Federation (IDF) consensus [22]. Exclusion criteria were previous cardiovascular disease, congestive heart failure, liver diseases (other than NAFLD), cancer or a history of malignancy in the previous 5 years, previous bariatric surgery, acute febrile illnesses, urinary tract infections, post-renal hematuria, hemochromatosis, protein overload, non-medicated depression or anxiety, alcohol and drug abuse, pregnancy, primary endocrinological diseases (other than hypothyroidism), concomitant therapy with steroids, intense physical exercise, or being unable to provide informed consent. At inclusion, all participants had a stable body weight and caloric intake during the previous six months. The current cross-sectional secondary analysis used a subset of 77 participants within the original study, with available data on hepatic iron load by MRI. Patients’ clinical histories were further revised to exclude patients with hereditary or acquired hemochromatosis or hemosiderosis. Screening and selection criteria for study inclusion are illustrated in the study flow-chart (Figure 1).

### 2.2. Ethics

The study protocols followed the Declaration of Helsinki ethical standards, and all the procedures were approved from the Ethics Committee of the Balearic Islands (ref. IB 2251/14 PI) and from the Ethics Committee of the University of Navarra (ref. 054/2015mod2). All participants were informed of the purpose and the implications of the study, and all provided the written informed consent to participate. The trial was registered at ClinicalTrials.gov with registry number NCT04442620 (https://clinicaltrials.gov/ct2/show/NCT04442620; accessed 30 April 2021).

### 2.3. General Data

During an initial interview with the study dietician and study nurse, information on socioeconomic status, medical history, current use of medication, previous diseases, and smoking status was obtained from all participants. As for alcohol consumption, participants were asked how many alcoholic beverages they consumed in a week on average and responded either none, <7/week, or ≥7/week. Patients consuming ≥7 alcoholic beverages on average a week were excluded if they presented with a drinking problem and/or a diagnosis of alcohol use disorder. Physical activity over the previous 12 months was also recorded using the validated Spanish version of the Minnesota Leisure Time Physical Activity Questionnaire, which estimates physical activity expressed as metabolic equivalents of tasks (METs·min·wk^−1^) [23,24].

### 2.4. Anthropometric Measurements

To minimize interobserver variability, personnel were kept to a minimum and trained to follow standard procedures. Weight (kg) and total body fat (kg and %) were measured using a Segmental Body Composition Analyzer for impedance testing (Tanita MC780P-MA, Tanita, Tokyo, Japan) with the subject wearing no shoes and light clothes (for which 0.6 Kg was subtracted from the total). Height was measured using a mobile stadiometer (Seca 213, SECA Deutschland, Hamburg, Germany) with the subject standing upright wearing no shoes, and with the head in the Frankfort horizontal plane. Body mass index (BMI) was calculated using the standard formula (weight in kilograms divided by the square of height in meters). Waist circumference (WC) was measured twice using an anthropometric tape in the standing position, midway between the last rib and the iliac crest. The mean of the two measurements was recorded to nearest millimeter. Blood pressure was measured in triplicate using a validated semi-automatic oscillometer (Omron HEM-705CP) with the patient sitting after 5 min rest. The mean of the three measurements, 2 min apart, was recorded.

### 2.5. Adherence to the Mediterranean Diet

During the baseline interview participants were also administered a 17-item Mediterranean diet questionnaire, a modified version of the previously validated questionnaire used in the PREDIMED trial [25], designed to assess adherence to the Mediterranean diet. Compliance with each item, relating to a specific food habit, was scored with 1 point and 0 points otherwise. Thus, the total score range was 0–17, with 0 meaning no adherence and 17 meaning maximum adherence.

### 2.6. Blood and Urine Sample Collection

Venous blood samples from all participants were drawn from the antecubital vein after nighttime fasting (12h) into one EDTA sample tube and one citrate sample tube (for plasma) and one serum sample tube before immediate centrifugation at 3000 rpm for 10 min. A single spot urine specimen collected in the early morning was requested from each participant.

### 2.7. Blood and Urine Sample Analysis

Fasting glycemia, glycosylated hemoglobin (Hb1Ac), bilirubin, aspartate aminotransferase (AST), alanine aminotransferase (ALT), gamma-glutamyl transferase (GGT), uric acid, transferrin, urea, creatinine, albumin, creatine kinase (CK), C-reactive protein (CRP), and the lipid status parameters (total cholesterol, high-density lipoprotein cholesterol (HDL-C), and triglyceride (TG)) were measured in serum on the Abbott ARCHITECT c16000 employing commercial kits (Abbott Diagnostics, Illinois, USA). The low-density lipoprotein cholesterol (LDL-C) was calculated according to the Friedewald formula. The serum ferritin and thyroid-stimulating hormone (TSH) were determined in an Abbott ARCHITECT i2000 using a chemiluminescence assay. The serum fasting insulin was measured on the Cobas e411 platform (Roche, Switzerland) using an enzyme-based electrochemiluminescence assay. Hematological parameters (hematocrit) and cell counts (erythrocytes, leukocytes, and platelets) were analyzed in whole blood in an automatic flow cytometer analyzer (Cell-Dyn Sapphire platform, Abbott Diagnostics, US), and the prothrombin time was analyzed in an ACL-TOP 700, (Instrumentation Laboratory, Bedford, US).

Urinary albumin excretion was measured from an early-morning urine sample as the urine albumin:creatinine ratio (UACR). The urinary albumin concentration was determined by immunoturbidimetric assay and urinary creatinine concentration was measured by a modified Jaffe method on an Abbott ARCHITECT c16000. The insulin resistance index was calculated using the Homeostatic Model Assessment for Insulin Resistance (HOMA-IR) formula by Matthews et al. [26]; eGFR was calculated using both the MDRD and the CKD-EPI formulas [27,28].

### 2.8. Diagnosis of NAFLD

Mean liver fat (%) was measured by MRI (Signa Explorer 1.5T, General Electric Healthcare, Chicago, IL, USA). The protocol for assessment of liver fat included the iterative decomposition of water and fat with echo asymmetry and the least-squares estimation quantitation (IDEAL IQ) sequence. Liver fat percentage was measured following liver image acquisition of 6 ROIs (regions of interest) of the liver parenchyma. NAFLD was staged as 1—mild (6.4%–17.4%), 2—moderate (17.4%–22.1%), and 3—severe ( >22.1%), according to Tang et al. [29]. Biochemical liver iron content (m/s and mg/g) was also measured by abdominal MRI, through the acquisition of images representing variations of T2* weighting. T2* mean values in ms, mg/g, and µmol/g were obtained by positioning 2 ROIs over both the left and right lobes. The hepatic iron load determined by T2*-weighted sequences was classified as normal (>20 ms), mild-moderate (10–20 ms), and severe (<10 ms) [30].

Evaluation of liver fibrosis by measurement of liver stiffness was determined by shear wave measurement (SWM) using the echographer Arietta V70 (Hitachi Medical System Europe Holding AG, Steinhausen, Switzerland). The patient was examined lying down in a resting respiratory position, with the right arm elevated above the head for optimal intercostal access. Measurements were obtained at least 1.5 to 2.0 cm deep to the Glisson capsule and at a depth of less than 4 cm to the skin surface to avoid reverberation artefact. Sonographers placed a region of interest (ROI) in the hepatic parenchyma, avoiding large blood vessels. The shear wave method works on generating shear waves in response to the focused ultrasound pulse. The shear wave propagation velocity value (Vs) obtained in the ROI was transformed into a quantitative measure of tissue stiffness, expressed in kilopascal (kPa). To quantitatively evaluate measurement reliability, the percentage of the measurements correctly detected is displayed as vs. effective rate (VsN). When a measurement from a series was exceptionally out-of-range or the VsN was below 20%, that value was discarded. The study was completed when a total of 10 valid measurements were obtained. Fibrosis was staged according to the METAVIR scoring system as no or mild fibrosis (F0–F1), moderate fibrosis (F2), severe fibrosis (F3), and cirrhosis (F4) [31]; cut-off values for F2, F3, and F4 were 6.44, 7.82, and 8.40 kPa, respectively [32].

### 2.9. Statistical Analysis

Sample size for the original trial was estimated using weight loss as the main prespecified outcome variable assuming a two-group (group ratio = 2) t-test (two-sided) of the difference between the control group and the two intervention groups. Currently, AASLD recommendations for amelioration of NAFLD are based on weight loss [19]. In accordance with previous evidence [33,34], a weight reduction difference of 2.5 kg with a SD of 4.5 was predicted between the intervention groups and the control group. On the basis of these assumptions, a total sample size of 150 patients would give the trial a 95% power to detect a statistically significant (α = 0.05) expected difference in weight between the CD and MD groups and account for a 20% drop-out rate. On the other hand, the present secondary analysis including a minimum of 73 patients would give the study a 90% power to accept the hypothesis of the relationship between HepFe and UACR assuming a two-tailed α error of 0.05.

Analyses were performed using the SPSS statistical software package version 25.0 (SPSS Inc., Chicago, IL, USA). Data are expressed as mean ± SD and median (interquartile range) for continuous variables and as counts (percentages) for categorical variables.

The normality assumption for continuous data was assessed with the Shapiro–Wilk test and visual inspection of histograms and scatter plots. Fasting insulin, HOMA-IR, and UACR presented a skewed distribution and were log-transformed before analysis; however, in the tables, they are presented as untransformed data for ease of interpretation.

Bivariate correlations between UACR and clinical variables were evaluated by Pearson’s correlation coefficient. The variables with a level of significance (*p*) below 0.05 (two-tailed) were regarded as potential covariates and entered in the multiple regression model to identify independent determinants of continuously modelled UACR. In the case of covariates presenting a high bivariate correlation (r ≥ 0.9), selection was guided by the strength and quality of the relationship and by clinical criteria. In the first model, only significant covariates were included; in the second model, covariates were adjusted for age, sex, eGFR, HbA1c, diabetes (y/n), stages of NAFLD, and fibrosis (y/n).

Lastly, the Welch’s t-test was used to account for unequal variances and unequal sample sizes when comparing differences in the mean of specific variables between categories of HepFe and NAFLD. Due to the small number of subjects in the higher categories of HepFe, patients in the mild/moderate and severe stages were grouped together and analyzed against patients with normal levels of HepFe. Similarly, due to the same reason, patients in the moderate and severe stages of NAFLD were grouped together and compared against patients in the mild stage of NAFLD.

All *p*-values were two-tailed, with *p* < 0.05.

## 3. Results

Of the 77 patients included in the secondary analysis, 2 were using long-acting insulin therapy for diabetes control. Since long-acting insulin might influence fasting insulin measures and HOMA-IR, these two patients were excluded from the analysis to avoid biased parameter estimates and biased test statistics and statistical significances. Finally, data from a total of 75 participants were analyzed (Figure 1).

General characteristics of the study population are displayed in Table 1. Table 2 showed anthropometric, clinical, and metabolic characteristics. Hepatic and renal characteristics are displayed in Table 3. The current analyses included 75 patients, 30 (40%) were women, 6 (8%) were current smokers, and 12 (16%) consumed ≥7 alcoholic drinks a week. The mean ± SD age was 52.7 ± 6.7 years. Mean BMI was 33.6 ± 4.0 kg/m^2^ and the mean WC was 112.9 ± 8.8 cm. Additionally, 43 participants (57.3%) had a diagnosis of high BP and 31 (41.3%) were taking antihypertensive medications, mainly ACE (angiotensin-converting enzyme) inhibitors or ARBs (Angiotensin II receptor blockers) (37.3%). Mean systolic blood pressure was 137.3 ± 16.8 mmHg and mean diastolic blood pressure was 82.6 ± 9.3 mmHg.

Overall, 20 participants (26.7%) had a diagnosis of Type 2 Diabetes Mellitus (T2DM) and 18 (24%) were being treated with oral hypoglycemic agents. Mean fasting glycemia for the whole sample was 114.1 ± 44.1 mg/dL, mean HbA1c was 6.0 ± 1.2%, mean fasting insulin was 19.5 ± 9.5 µIU/L, and mean HOMA-IR was 5.6 ± 3.4.

Serum ferritin averaged at 155.53 ± 156.56 ng/mL. Mean percentage hepatic fat as measured by MRI was 15.9 ± 9.9. The prevalence of moderate (stage 2) and severe (stage 3) stages of NAFLD was 12% and 22.7%, respectively, with most patients (65.3%) presenting a stage 1 (mild) NAFLD. The degree of HepFe was rarely above a moderate degree and never above stage 3; the prevalence of mild/moderate and severe hepatic iron load were 6.7% and 2.7%, respectively. Only a small percentage of patients suffered from fibrosis (mild, moderate, and severe fibrosis were respectively 9.3%, 1.3%, and 5.3%), while the majority (73.3%) did not present clinically significant levels of liver fibrosis. Mean scores of transaminase enzymes were within normal ranges, with AST averaging 27.1 ± 14.6 IU/L, ALT averaging 38.4 ± 34.7 IU/L, and GGT 58.4 ± 71.5 IU/L.

The mean ± SD eGFR was 91.7 ± 14.2 and 92.3 ± 17.9 mL/min/1.73 m^2^ using the MDRD and CKD-EPI formulas, respectively. Finally, the prevalence of UACR > 30 mg/g and in the high normal range (10–29 mg/g) was 8% and 26%, respectively. The average UACR was 11.3 ± 17.5 mg/g.

Table 4 shows predictors of UACR at multivariate analysis after controlling for age, gender, and other potential confounders. Pearson’s correlation analyses suggested that UACR correlated with mean liver fat % by RMN, TG, HepFe, serum ferritin, fasting insulin, HOMA-IR, and platelets (*p* > 0.05). Multiple linear regression analysis further revealed that HepFe, serum ferritin, and platelets were associated with mean UACR (R^2^ = 0.319; *p* < 0.001). After adjusting for gender, age, eGFR, HbA1c, T2DM (y/n), stages of NAFLD (1/2/3), and liver fibrosis (y/n), it was found that HepFe (*p* = 0.020), serum ferritin (*p* = 0.040), fasting insulin (*p* = 0.049), and platelets (*p* = 0.009) were significantly associated with mean UACR (R^2^ = 0.370; *p* = 0.007).

**Figure 2:**
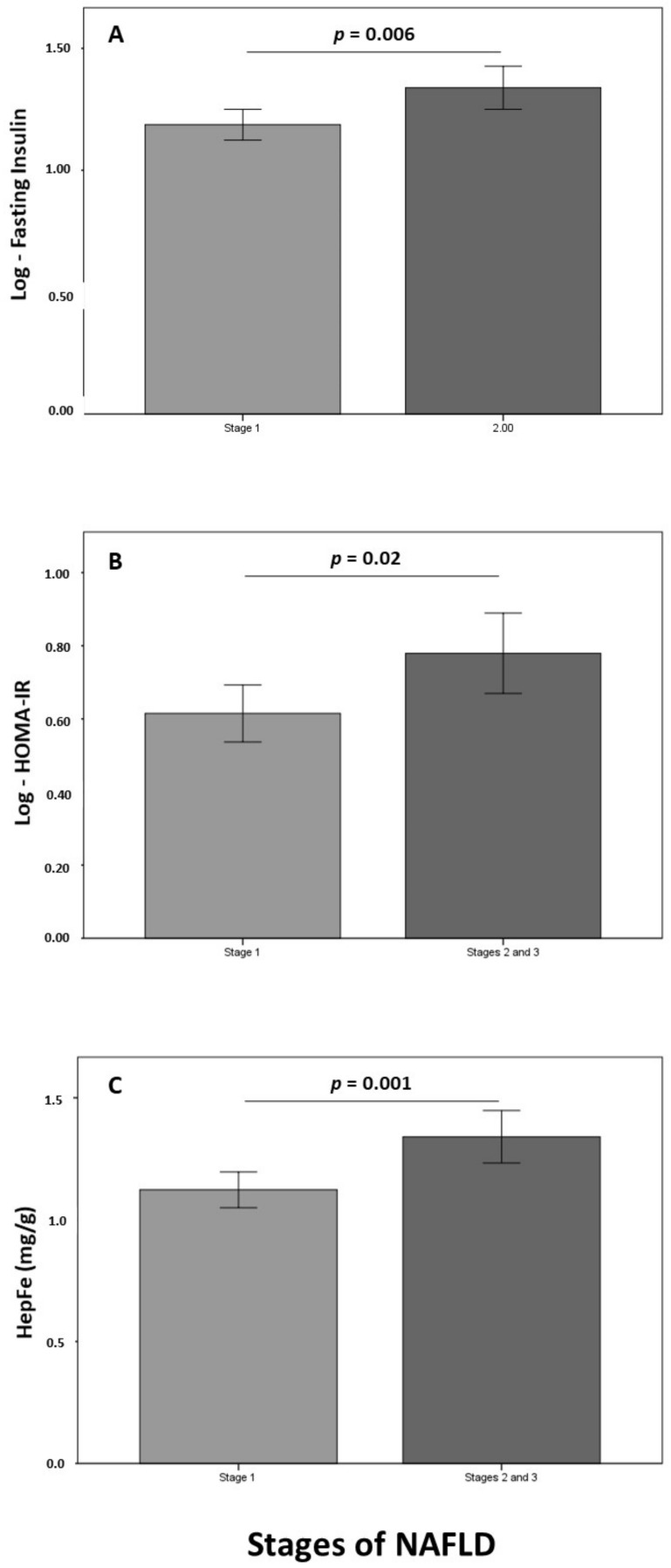
Differences in fasting insulin (**A**), HOMA-IR (**B**), and HepFe (**C**) between stage 1 and stages 2 and 3 of NAFLD.

Figure 2 shows differences in fasting insulin (Figure 2A), HOMA-IR (Figure 2B), and HepFe (Figure 2C) between the two categories of NAFLD. Fasting insulin, HOMA-IR, and HepFe were higher in patients in stages 2 and 3 of NAFLD as compared to stage 1 (*p* = 0.006, *p* = 0.020, and *p* = 0.001, respectively). Systolic blood pressure was also higher in patients in stages 2 and 3 of NAFLD (143.7 ± 17.8) as compared to stage 1 (134.0 ± 15.5) (*p* = 0.030, data not shown). No differences in UACR, platelets, or serum ferritin were observed across stages of NAFLD.

Table 5 shows characteristics of the MetS, hepatic, and renal variables of the study population according to stages of HepFe. Between the categories of HepFe, the UACR, serum ferritin, HOMA-IR, and NAFLD (mean fat%) were higher in the mild to severe category as compared to the normal range.

## 4. Discussion

The aim of the present study was to explore whether liver iron accumulation independent of HHC was associated with albuminuria in patients with NAFLD, on the basis that conditions such as hyperferritinemia, insulin resistance, and NAFLD, which have been previously observed to be independently related to liver iron accumulation, also contribute to albuminuria.

The current study found that liver iron accumulation was indeed independently associated with the mean UACR. Moreover, fasting insulin, serum ferritin, and platelets, but not liver steatosis, were also associated with the mean UACR.

Further findings were that the UACR increased between stage 0 and stages 1–2 of HepFe; patients with higher levels of HepFe showed higher levels of NAFLD, serum ferritin, and HOMA-IR; and, finally, patients in stage 2 and 3 of NAFLD showed higher levels of SBP, insulin, HOMA-IR, and HepFe, as compared to patients in stage 1.

To the best of our knowledge, there are not available studies so far with evidence that hepatic iron load is significantly associated with albuminuria in patients with NAFLD and MetS. The association between hepatic iron load and albuminuria may lay upon their related features. Unexplained hepatic iron overload is strongly related to insulin resistance and hyperferritinemia in non-C282Y-homozygous patients [35], and it increases from early to moderate NAFLD such that it may be involved in its pathogenesis [36]. Moreover, defined as a condition called DIOS, even at mild levels of accumulation, hepatic iron load is related to NAFLD, hyperferritinemia, fasting insulin, insulin resistance, and other traits of the MetS [3,37,38,39]. In turn, such conditions are also independently associated with albuminuria [8,9,10,11,40]. Accordingly, in the current study, patients with stages 1–2 of hepatic iron load presented significantly higher levels of hepatic fat percentage, serum ferritin, insulin resistance, and UACR, than in patients at stage 0. Although very limited, some evidence could support our findings. A small group of hyper-transfused patients with beta-thalassemia presenting iron overload experienced an early and accelerated course of diabetic nephropathy [41]. The authors suggested that increased reactive oxygen species activity derived from iron accumulation was the main determinant of the observed progressive microalbuminuria. The pro-oxidant capacity of iron overload has been demonstrated to have a role in the development and progression of chronic kidney disease, such that iron treatment among patients with end-stage renal disease or hemodialysis needs to be done with caution [42]. Dietary iron restriction halted progression of diabetic nephropathy in db/db mice, and, importantly, it did so partly due to reduced oxidative stress [43]. Similarly, in a small sample of patients with diabetic nephropathy, treatment with an iron chelator reduced albumin levels over a 9-month period [44].

The current findings that albuminuria is not associated with NAFLD differ from previous evidence demonstrating a causal relationship. In a recent metanalysis [17], it was concluded that, among NAFLD patients, the risk of developing increased albuminuria (UACR ≥ 30 mg/g) was significantly higher than in patients without NAFLD (pooled odds ratio (OR) of 1.67 (95% confidence interval: 1.32–2.11)). Present results showed no significant relationship between UACR and NAFLD at multivariate regression; also, no differences in UACR were found between categories of NAFLD. This may be due to the limited number of cases in the current sample and that most patients showed normal levels of albuminuria (UACR < 30 mg/g), making it difficult to explore the relationship between albuminuria and NAFLD at clinically relevant stages of urinary protein excretion. Moreover, most studies included in the metanalysis [17] used ultrasonography to diagnose NAFLD; although such a technique is non-invasive, safe, inexpensive, and easy to perform, its results are operator and equipment dependent, and its sensitivity is known to be reduced in centrally obese subjects [45]. In contrast, the current study used the MRI technique, which is currently the most accurate non-invasive modality for NAFLD evaluation. The small sample and differences in levels of UACR and imaging techniques may account for variations in the observations.

The association between fasting insulin with renal outcomes in diabetic as well as non-diabetic patients has already been observed in previous studies [46], especially if increased fasting insulin is considered as an early marker of insulin resistance [47,48]. Hyperinsulinemia may induce increased albuminuria through its direct effect on glomerular hyperfiltration, endothelial dysfunction, and increased vascular permeability over the long term [36], although in non-diabetic subjects, even a short-term insulin infusion increases urinary albumin excretion [49]. On the other hand, chronically increased albuminuria is also strongly related to higher levels of fasting insulin, independent of age, sex, BMI, and blood pressure [50].

In the present study, serum ferritin resulted as being significantly related to mean albuminuria in our patients. This finding is in line with previous studies reporting that serum ferritin, a good indicator of iron stores the body [51] and an early marker of insulin resistance [6,7], correlates with other components of the MetS [52] including increased albuminuria [8,9,10] and overt proteinuria [53]. Indeed, when increased body iron stores are reduced in NAFLD, either through a controlled iron dietary regime or bloodletting, insulin resistance is improved [54,55,56]. In a randomized study on carbohydrate-intolerant patients, iron depletion to near iron deficiency was associated with a decrease in both fasting and glucose-stimulated plasma insulin concentrations in a subgroup of 17 subjects with hyperferritinemia and non-biopsy-proven NAFLD [55]. In another controlled randomized trial of 28 diabetic patients with high serum ferritin, bloodletting improved both insulin sensitivity and beta cell function but had no significant effect on blood glucose [56]. Interestingly, iron depletion through a diet low in red meat was also observed to reduce albuminuria in 17 T2DM patients with macroalbuminuria (24 h urinary excretion rate ≥200 μg/min) [54]; unfortunately, the study does not mention whether insulin resistance was concomitantly reduced.

In the current study, platelet count was also significantly related with mean UACR. Previous studies on establishing early non-invasive detectors of NAFLD found that increased platelet count is higher in patients with liver fibrosis as compared to those without [57,58], such that platelet count is included as an operand in the formula to calculate the NAFLD fibrosis score, estimating presence of advanced fibrosis [59]. On the other hand, previous studies on the association between platelets and UACR are very few; nevertheless, they show an independent relationship between mean platelet volume, increased albuminuria, and other microvascular complications such as retinopathy in T2DM [60,61]. Platelet activity plays a major role in the pathogenesis of endothelial injury and vascular disease [62]; moreover, it has been associated with hyperglycemia [63] and insulin resistance [64,65,66], which in turn are associated with increased albuminuria [11]. Although platelet count seems to share common pathways to other risk factors associated with increased albuminuria, in the current analysis, such association was independent of insulin resistance, diabetes, liver fibrosis, and other potential confounders.

Finally, when exploring differences in metabolic and renal variables across stages of NAFLD, patients in the higher stages of the disease showed higher levels of hepatic iron load and fasting insulin, and higher scores of HOMA-IR, as compared to patients in stage 1. Accordingly, increased hepatic iron stores are observed in about half of adult NAFLD patients and may potentiate the onset and progression of disease by altering insulin signaling and metabolic function [2,67]. Results from the National Hemochromatosis Transplant Registry (NHTR) showed that, even in the absence of HHC, iron overload was recurring in patients with liver disease and it significantly associated with hepatocellular carcinoma, even after adjusting for the underlying etiology of liver disease [68]. Similarly, NAFLD has been associated with increased insulin resistance compared with controls, even in the absence of obesity, and that insulin resistance increased with increasing degrees of steatosis [69,70,71,72,73,74,75]. Current findings along with existing evidence highlight the importance of recognizing NAFLD as a progressive disease strongly influenced by metabolic abnormalities. A recent meta-analysis including both diabetic and non-diabetic patients associated the presence and severity of NAFLD with an increased risk and severity of CKD [76], which is also strongly associated with metabolic dysfunction [77].

NAFLD, hepatic iron load, and metabolic abnormalities such as increased fasting insulin, insulin resistance, and platelet count might be linked together, and treating patients at early stages might prevent extrahepatic complications as well as the adverse clinical outcomes.

## 5. Strengths and Limitations

The main strength of this study is that a diagnosis of NAFLD was obtained by MRI, which is considered the most sensitive and accurate non-invasive method for quantifying liver fat [29,39,51]. The limitations are linked to selection bias, the limited number of patients, and to the design of the study, which lacks a control group. Selection bias could be hypothesized as the dataset was created according to available data on HepFe. By not knowing the levels of hepatic iron in the excluded population, results might over- or underestimate the true risk [77]. Finally, a bigger sample including patients with higher levels of albuminuria and hepatic iron load, compared to a control group of patients with NAFLD without hepatic iron load, could give a more confident answer to the possible relationship between the liver and kidney mediated by liver iron accumulation in patients with MetS.

## 6. Conclusions

This aim of the present secondary analysis was to assess whether a relationship exists between hepatic iron load and UACR in the context of DIOS and NAFLD. The current findings showed that hepatic iron load, fasting insulin, serum ferritin, and platelets were associated with mean albuminuria independently of gender, age, diabetes, HbA1c, stages of NAFLD, and presence of liver fibrosis.

Increased hepatic and body stores of iron have been linked to increased risk of metabolic complications and progression of hepatic and cardiovascular disease, among others. Excessive iron might play a direct effect on insulin resistance, and the DIOS condition may accelerate the evolution to T2DM, cardiovascular disease, and liver disease. In turn, excessive iron might further expose patients with confirmed NAFLD to other metabolic complications such as increased albuminuria.

On this basis of previous observations and current results, the association between DIOS, MetS, and NAFLD may represent a new risk factor for the development of increased albuminuria. This new finding is relevant to the field of renal involvement in metabolic conditions as it contributes to expand the knowledge of the connection between NAFLD and chronic kidney disease. Most importantly, DIOS, MetS, NAFLD, and increased albuminuria are treatable conditions if approached during the early stages. The present results show a potential effect of HepFe and ferritin on UACR, in the context of NAFLD and MetS, at levels below clinical significance in a relatively young population with normal renal function and free of cardiovascular and other diabetic complications. Early treatment of iron accumulation could be included as a preventative strategy in patients with NAFLD and MetS. In controlled studies in NAFLD, bloodletting or a low-iron diet improved insulin resistance and reduced plasma levels of insulin. Further evidence produced by randomized controlled studies is needed to assess whether iron depletion can also modify renal outcomes.

## Figures and Tables

**Table 1:** General characteristics of the current sample (*n* = 75).

Female	30 (40.0%)
Who were having menopause	19 (25.3%)
Age (y) (mean ± SD)	52.7 ± 6.7 (6.86)
Currently smoking	6 (8.0%)
Alcohol ≥7 drinks/week	12 (16.0%)
Regular physical activity	
None	34 (45.3%)
Light	27 (36.0%)
Moderate	12 (16.0%)
Heavy	2 (2.7%)
Type 2 diabetes	20 (26.7%)
Hypertension	43 (57.3%)
Concomitant medications	
Hypoglycemic agents	
Oral hypoglycemic agents alone	18 (24.0%)
Antihypertensive agents	
Any	31 (41.3%)
Diuretics	12 (16.0%)
μ-blockers	5 (6.7%)
Calcium-channel blockers	4 (5.3%)
ACE inhibitors, ARBs	28 (37.3%)
Lipid-lowering agents	
Any	23 (30.7%)
Statins alone	14 (18.7%)
Fibrate alone	6 (8.0%)
Statins and fibrate	3 (4.0%)
Antiplatelet agents	3 (4.0%)
Concomitant medications—other	
Any	49 (65.3%)
Thyroid medications	6 (8%)
Depression/anxiety/insomnia	14 (18.7%)
Gout medications	9 (12.0%)

Data are expressed as counts (percentages %), unless otherwise stated. Abbreviations: ACE = angiotensin-converting enzyme; ARBs = Angiotensin II receptor blockers; SD = standard deviation.

**Table 2:** Anthropometric, clinical, and metabolic characteristics of the current study population (*n* = 75).

	Mean ± SD	Median (IQR)
Anthropometric variables		
Weight (kg)	94.1 ± 13.7	92.2 (18.6)
BMI (kg/m^2^)	33.6 ± 4.0	32.6 (6.0)
Waist circumference (cm)	112.9 ± 8.8	111.3 (14.6)
Total body fat (kg)	32.7 ± 8.0	30.3 (9.2)
Total body fat (%)	34.7 ± 6.7	33.4 (11.4)
Clinical parameters		
Systolic BP (mmHg)	137.3 ± 16.8	136.3 (20.2)
Diastolic BP (mmHg)	82.6 ± 9.3	80.7 (13.4)
MAP (mmHg)	100.9 ± 11.1	99.0 (13.6)
HR (bpm)	70.6 ± 10.8	70.2 (14.1)
Metabolic variables		
Glycemia (mg/dL)	114.1 ± 44.1	105.0 (23.0)
HbA1c (%)	6.0 ± 1.2	5.7 (0.6)
Fasting insulin (µIU/L)	19.5 ± 9.5	17.8 (12.7)
HOMA-IR	5.6 ± 3.4	5.1 (4.5)
Blood lipids		
Total cholesterol (mg/dL)	210.0 ± 41.5	210.0 (58.0)
HDL cholesterol (mg/dL)	42.4 ± 8.7	41.0 (9.0)
LDL cholesterol (mg/dL)	128.1 ± 32.5	127.0 (44.7)
VLDL cholesterol (mg/dL)	41.0 ± 30.7	35.0 (17.0)
Triglycerides (mg/dL)	203.4 ± 152.1	175.0 (86.0)
Physical Activity		
METs (min/week)	3072.2 ± 3641.2	1920.0 (2680.2)
Mediterranean Diet Adherence	8.1 ± 2.7	8.0 (4.0)
Other variables		
Uric acid (mg/dL)	6.3 ± 1.5	6.3 (1.8)
Serum Ferritin (ng/mL)	155.5 ± 156.6	98.0 (138.0)
Transferrin (mg/dL)	271.5 ± 38.0	268.5 (42.7)
CK (U/L)	149.9 ± 161.4	120.0 (91.0)
PCR (mg/dL)	0.5 ± 0.6	0.3 (0.5)
TSH (mUI/L)	2.02± 1.1	1.8 (1.3)

Data are expressed as mean ± standard deviation and median (interquartile range). Abbreviations: BMI = body mass index; BP = blood pressure; HR = heart rate; MAP = mean arterial pressure; METs = metabolic equivalents.

**Table 3:** Hepatic and renal characteristics of the current study population (*n* = 75).

	Mean ± SD	Median (IQR)
Hepatic variables		
Stages of NAFLD		
1: Mild (6.4%–17.4%)		49 (65.3%)
2: Moderate (17.4%–22.1%)		9 (12.0%)
3: Severe (>22.1%)		17 (22.7%)
NAFLD—Mean liver fat %	15.9 ± 9.9	12.4 (11.3)
Stages of HepFe (ms)		
0: Normal (>20 ms)		68 (90.7%)
1: Mild/moderate (10–20 ms)		5 (6.7%)
2: Severe (<10 ms)		2 (2.7%)
Hepatic iron load (mg/g)	1.2 ± 0.3	1.1 (0.3)
Hepatic iron load (ms)	27.0 ± 5.5	26.9 (7.1)
Stages of fibrosis		
F0–F1: Normal (<6.44 kPa)		55 (73.3%)
F2: Mild (6.44–7.81 kPa)		7 (9.3%)
F3: Moderate (7.82–8.39 kPa)		1 (1.3%)
F4: Severe (>8.39 kPa)		4 (5.3%)
Fibrosis (kPa)	5.2 ± 1.5	4.90 (1.80)
Liver function test		
AST (IU/L)	27.1 ± 14.6	23.0 (11.50)
ALT (IU/L)	38.4 ± 34.7	31.0 (25.0)
GGT (IU/L)	58.4 ± 71.5	38.0 (28.0)
Total bilirubin (mg/dL)	0.71± 0.4	0.60 (0.35)
Platelets (10^3^/uL)	238.4 ± 46.8	240.0 (63.0)
Kidney function		
MDRD (ml/min/1.73 m^2^)	91.7 ± 14.2	94.4 (18.9)
CKD-EPI (ml/min/1.73 m^2^)	92.3 ± 17.9	98.8 (16.7)
Serum albumin (g/L)	4.4 ± 0.4	4.4 (0.3)
Serum creatinine (mg/dL)	0.8 ± 0.2	0.8 (0.1)
Urea (mg/dL)	37.3 ± 10.6	34.0 (15.0)
Urine albumin (mg/L)	13.4 ± 21.4	6.0 (11.5)
Urine creatinine (mg/L)	121.7 ± 48.5	116.9 (64.8)
UACR (mg/g)	11.3 ± 17.5	5.6 (7.75)
Albuminuria high normal (10–29 mg/g)		20 (26.0%)
Increased albuminuria (>30 mg/g)		6 (8.0%)

Data are expressed as mean ± standard deviation, and median (interquartile range) for continuous variables, and as counts (percentages %) for categorical variables. Abbreviations: HepFe = hepatic iron load; NAFLD = non-alcoholic fatty liver disease; UACR = urine albumin-to-creatinine ratio.

**Table 4:** Predictors of UACR at multivariate analysis after controlling for confounding variables in patients with non-alcoholic fatty liver disease and metabolic syndrome.

	*b* (95%CI)	Std. Error	β	*p*
Model 1—Variables				
(Constant)	−1.48 (−2.63–(−0.32))	0.58		0.010
NAFLD—Mean liver fat %	0.00 (−0.01–0.01)	0.01	−0.09	0.490
Triglycerides (mg/dL)	0.00 (0.00–0.00)	0.00	0.04	0.740
Hepatic iron load (mg/g)	0.43 (0.02–0.84)	0.20	0.30	0.040
Serum Ferritin (ng/mL)	0.00 (0.00–0.00)	0.00	0.33	0.020
Fasting insulin (µIU/L) ^#^	1.00 (−0.01–2.01)	0.51	0.55	0.050
HOMA-IR ^#^	−0.60 (−1.46–0.26)	0.43	−0.40	0.170
Platelets (10^3^/uL)	0.00 (0.00–0.01)	0.00	0.48	< 0.001
Model 2—Variables				
(Constant)	−4.26 (−7.95–(−0.58))	1.84		0.020
NAFLD—Mean liver fat %	0.00 (−0.02–0.02)	0.01	0.11	0.660
Triglycerides (mg/dL)	0.00 (0.00–0.00)	0.00	0.01	0.920
Hepatic iron load (mg/g)	0.54 (0.08–1.00)	0.23	0.38	0.020
Serum Ferritin (ng/mL)	0.00 (0.00–0.00)	0.00	0.34	0.040
Fasting insulin (µIU/L) ^#^	2.60 (0.01–5.18)	1.29	1.43	0.049
HOMA-IR ^#^	−2.10 (−4.53–0.33)	1.21	−1.41	0.090
Platelets (10^3^/µL)	0.00 (0.00–0.01)	0.00	0.39	0.009
Gender (m/f)	0.05 (−0.16–0.26)	0.10	0.07	0.620
Age (y)	0.01 (−0.01–0.03)	0.01	0.17	0.230
eGFR (ml/min/1.73 m^2^)	0.00 (0.00–0.01)	0.00	0.20	0.150
HbA1c (%)	0.16 (−0.05–0.37)	0.11	0.51	0.140
T2DM (y/n)	−0.10 (−0.36–0.16)	0.13	−0.12	0.430
Stage of NAFLD (1/2/3)	−0.08 (−0.31–0.15)	0.11	−0.18	0.480
Liver fibrosis (y/n)	−0.01 (−0.25–0.23)	0.12	−0.01	0.920

R^2^ = 0.319 for Step 1 (*p*< 0.001); R^2^ = 0.370 for Step 2 (*p* = 0.007); R^2^Δ = 0.051 (*p* = 0.68). The table reports the following: unstandardized B (b), confidence intervals (95% CI) and standard errors (Std. Error), the standardized coefficient Beta (β), and the significance level of each variable (*p*) as predictors of UACR (urine albumin-to-creatinine ratio); # = log-transformed.

**Table 5:** Characteristics of the MetS, hepatic, and renal variables of the study population according to the stages of HepFe.

	Stage 0 (*n* = 68)	Stage 1–2 (*n* = 7)	
	Mean ± SD	Mean ± SD	*p*
Weight (kg)	93.5 ± 13.5	101.0 ± 15.3	0.290
BMI (kg/m^2^)	33.4 ± 4.0	34.7 ± 3.5	0.440
Waist circumference (cm)	112.6 ± 8.7	115.8 ± 9.9	0.480
Systolic BP (mmHg)	136.2 ± 16.6	149.7 ± 15.8	0.090
Diastolic BP (mmHg)	81.7 ± 8.7	92.0 ± 10.4	0.060
MAP (mmHg)	99.9 ± 10.6	111.2± 11.8	0.070
HR (bpm)	70.1 ± 10.0	75.5 ± 17.5	0.490
Blood glucose (mg/dL)	112.2 ± 44.2	132.3 ± 41.6	0.260
HbA1c (%)	6.0 ± 1.3	6.1 ± 1.1	0.910
Fasting insulin (µIU/L)^#^	19.0 ± 9.4	25.6 ± 9.2	0.130
HOMA-IR ^#^	5.3 ± 3.1	9.5 ± 5.4	0.040
Serum ferritin (ng/mL)	125.1 ± 114.4	495.0 ± 172.1	0.003
HDL-cholesterol (mg/dL)	42.6 ± 9.0	40.1 ± 3.3	0.160
Triglycerides (mg/dL)	187.9 ± 97.7	353.1 ± 387.1	0.300
NAFLD (mean fat %)	14.2 ± 7.5	31.8 ± 16.2	0.030
Fibrosis (kPa)	5.1 ± 1.5	5.7 ± 1.0	0.200
HepFe (mg/g)	1.1 ± 0.1	1.8 ± 0.5	0.007
Platelets (10^3^/uL)	240.5 ± 45.7	218.7 ± 56.8	0.360
MDRD (ml/min/1.73 m^2^)	92.2 ± 13.7	86.2 ± 19.0	0.440
CKD-EPI (ml/min/1.73 m^2^)	92.9 ± 16.9	86.5 ± 26.8	0.560
UACR (mg/g) ^#^	8.2 ± 8.5	40.0 ± 42.1	0.020

Data are expressed as mean (standard deviation, SD). BMI = body mass index; BP = blood pressure; HR = heart rate; HepFe = hepatic iron load; MAP = mean arterial pressure; MetS = metabolic syndrome; NAFLD = non-alcoholic fatty liver disease; # = log-transformed.

## Data Availability

There are restrictions on the availability of data for this trial, due to the signed consent agreements around data sharing, which only allow access to external researchers for studies following the project purposes. Requestors wishing to access the trial data used in this study can make a request to pep.tur@uib.es.

## Clinical Trials Registration

The trial was registered at ClinicalTrials.gov with registry number NCT04442620 (https://clinicaltrials.gov/ct2/show/NCT04442620; accessed 30 April 2021).

## References

[1] Younossi Z.M. (2019). Non-alcoholic fatty liver disease—A global public health perspective. J. Hepatol..

[2] Jézéquel C., Lainé F., Laviolle B., Kiani A., Bardou-Jacquet E., Deugnier Y. (2015). Both hepatic and body iron stores are increased in dysmetabolic iron overload syndrome. A case-control study. PLoS ONE.

[3] Deugnier Y., Bardou-Jacquet É., Lainé F. (2017). Dysmetabolic iron overload syndrome (DIOS). Presse Med..

[4] Bugianesi E., Moscatiello S., Ciaravella M.F., Marchesini G. (2010). Insulin resistance in nonalcoholic fatty liver disease. Curr. Pharm. Des..

[5] Trombini P., Piperno A. (2007). Ferritin, metabolic syndrome and NAFLD: Elective attractions and dangerous liaisons. J. Hepatol..

[6] Haap M., Fritsche A., Mensing H.J., Häring H.U., Stumvoll M. (2003). Association of high serum ferritin concentration with glucose intolerance and insulin resistance in healthy people. Ann. Intern. Med..

[7] Sivasankari J., Thiruchelvan V. (2017). Serum Ferritin: An Early Marker of Insulin Resistance in Metabolic Syndrome. Int. J. Sci..

[8] Kim B.J., Kim B.S., Kang J.H. (2014). The association between serum ferritin level, microalbuminuria and non-alcoholic fatty liver disease in non-diabetic, non-hypertensive men. Clin. Exp. Hypertens.

[9] Amin R.F., El Bendary A.S., Ezzat S.E., Mohamed W.S. (2019). Serum Ferritin level, microalbuminuria and non-alcoholic fatty liver disease in type 2 diabetic patients. Diabetes Metab. Syndr..

[10] Hwang S.T., Cho Y.K., Yun J.W., Park J.H., Kim H.J., Park D.I., Sohn C.I., Jeon W.K., Kim B.I., Rhee E.J. (2010). Impact of nonalcoholic fatty liver disease on microalbuminuria in patients with prediabetes and diabetes. Intern. Med. J..

[11] Tuttle K.R. (2005). Renal manifestations of the metabolic syndrome. Nephrol. Dial. Transplant..

[12] Arnlöv J., Evans J.C., Meigs J.B., Wang T.J., Fox C.S., Levy D., Benjamin E.J., D’Agostino R.B., Vasan R.S. (2005). Low-grade albuminuria and incidence of cardiovascular disease events in nonhypertensive and nondiabetic individuals: The Framingham Heart Study. Circulation.

[13] Gerstein H.C., Mann J.F.E., Yi O., Zinman B., Dinneen S.F., Hoogwerf B., Hallé J.P., Young J., Rashkow A., Joyce C. (2001). Albuminuria and risk of cardiovascular events, death, and heart failure in diabetic and nondiabetic individuals. JAMA.

[14] Wachtell K., Ibsen H., Olsen M.H., Borch-Johnsen K., Lindholm L.H., Mogensen C.E., Dahlöf B., Devereux R.B., Beevers G., de Faire U. (2003). Albuminuria and cardiovascular risk in hypertensive patients with left ventricular hypertrophy: The LIFE study. Ann. Intern. Med..

[15] Klausen K., Borch-Johnsen K., Feldt-Rasmussen B., Jensen G., Clausen P., Scharling H., Appleyard M., Jensen J.S. (2004). Very low levels of microalbuminuria are associated with increased risk of coronary heart disease and death independently of renal function, hypertension, and diabetes. Circulation.

[16] Jessani S., Levey A.S., Chaturvedi N., Jafar T.H. (2012). High normal levels of albuminuria and risk of hypertension in Indo-Asian population. Nephrol. Dial. Transplant..

[17] Wijarnpreecha K., Thongprayoon C., Boonpheng B., Panjawatanan P., Sharma K., Ungprasert P., Pungpapong S., Cheungpasitporn W. (2018). Nonalcoholic fatty liver disease and albuminuria: A systematic review and meta-analysis. Eur. J. Gastroenterol. Hepatol..

[18] Abd El-Kader S.M., El-Den Ashmawy E.M. (2015). Non-alcoholic fatty liver disease: The diagnosis and management. World J. Hepatol..

[19] Chalasani N., Younossi Z., Lavine J.E., Diehl A.M., Brunt E.M., Cusi K., Charlton M., Sanyal A.J. (2012). The diagnosis and management of non-alcoholic fatty liver disease: Practice Guideline by the American AssociationAmerican Association for the Study of Liver Diseases, American College of Gastroenterology, and the Americanthe American Gastroenterological Association. Hepatology.

[20] Younes R., Bugianesi E. (2018). The Impact of Metabolic Syndrome on the Outcome of NASH: Cirrhosis, Hepatocellular Carcinoma, and Mortality. Curr. Hepatol. Rep..

[21] Lainé F., Bendavid C., Moirand R., Tessier S., Perrin M., Guillygomarc’h A., Guyader D., Calon E., Renault A., Brissot P. (2004). Prediction of liver fibrosis in patients with features of the metabolic syndrome regardless of alcohol consumption. Hepatology.

[22] The International Diabetic Federation (IDF) The IDF Consensus Worldwide De-Finition of Definition of the Metabolic Syndrome. http://www.idf.org/webdata/docs/IDF_Meta_def_final.pdf.

[23] Elosua R., García M., Aguilar A., Molina L., Covas M.I., Marrugat J. (2000). Validation of the Minnesota Leisure Time Physical Activity Questionnaire in Spanish Women. Med. Sci. Sports Exerc..

[24] Elosua R., Marrugat J., Molina L., Pons S., Pujol E. (1994). Validation of the Minnesota Leisure Time Physical Activity Questionnaire in Spanish Men. Am. J. Epidemiol..

[25] Schroder H., Fitó M., Estruch R., Martínez-González M.A., Corella D., Salas-Salvadó J., Lamuela-Raventós R., Ros E., Salaverría I., Fiol M. (2011). A short screener is valid for assessing Mediterranean diet adherence among older Spanish men and women. J. Nutr..

[26] Matthews D.R., Hosker J.P., Rudenski A.S., Naylor B.A., Treacher D.F., Turner R.C. (1985). Homeostasis model assessment: Insulin resistance and beta-cell function from fasting plasma glucose and insulin concentrations in man. Diabetologia.

[27] Levey A.S., Coresh J., Greene T., Stevens L.A., Zhang Y.L., Hendriksen S., Kusek J.W., Van Lente F. (2006). Using standardized serum creatinine values in the modification of diet in renal disease study equation for estimating glomerular filtration rate. Ann. Intern. Med..

[28] Levey A.S., Stevens L.A., Schmid C.H., Zhang Y.L., Castro 3rd A.F., Feldman H.I., Kusek J.W., Eggers P., Van Lente F., Greene T. (2009). A new equation to estimate glomerular filtration rate. Ann. Intern. Med..

[29] Tang A., Tan J., Sun M., Hamilton G., Bydder M., Wolfson T., Gamst A.C., Middleton M., Brunt E.M., Loomba R. (2013). Nonalcoholic fatty liver disease: MR imaging of liver proton density fat fraction to assess hepatic steatosis. Radiology.

[30] Chandarana H., Lim R.P., Jensen J.H., Hajdu C.H., Losada M., Babb J.S., Huffman S., Taouli B. (2009). Hepatic Iron Deposition in Patients With Liver Disease: Preliminary Experience With Breath-Hold Multiecho T2*-Weighted Sequence. Am. J. Roentgenol..

[31] The French METAVIR Cooperative Study Group (1994). Intraobserver and interobserver variations in liver biopsy interpretation in patients with chronic hepatitis C. Hepatology.

[32] Ferraioli G., Maiocchi L., Lissandrin R., De Silvestri A., Tinelli C., Filice C. Non-Invasive Staging of Liver Fibrosis in Patients with Chronic Viral Hepatitis: Performance of a Shear Wave Measurement Method. Poster No.: B-1248. ECR Congress 2017.

[33] De la Iglesia R., Lopez-Legarrea P., Abete I., Bondia-Pons I., Navas-Carretero S., Forga L., Martinez J.A., Zulet M.A. (2014). A new dietary strategy for long-term treatment of the metabolic syndrome is compared with the American Heart Association (AHA) guidelines: The MEtabolic Syndrome REduction in NAvarra (RESMENA) project. Br. J. Nutr..

[34] Rubio Herrera M.A. (2005). Evidence-based medicine: Nutrition in obesity. Endocrinol. Nutr..

[35] Mendler M.H., Turlin B., Moirand R., Jouanolle A.M., Sapey T., Guyader D., Le Gall J.Y., Brissot P., David V., Deugnier Y. (1999). Insulin resistance-associated hepatic iron overload. Gastroenterology.

[36] Ryan J.D., Armitage A.E., Cobbold J.F., Banerjee R., Borsani O., Dongiovanni P., Neubauer S., Morovat R., Wang L.M., Pasricha S.R. (2017). Hepatic iron is the major determinant of serum ferritin in NAFLD patients. Liver Int..

[37] Dongiovanni P., Fracanzani A.L., Fargion S., Valenti L. (2011). Iron in fatty liver and in the metabolic syndrome: A promising therapeutic target. J. Hepatol..

[38] Buzzetti E., Petta S., Manuguerra R., Luong T.V., Cabibi D., Corradini E., Craxì A., Pinzani M., Tsochatzis E., Pietrangelo A. (2019). Evaluating the association of serum ferritin and hepatic iron with disease severity in non-alcoholic fatty liver disease. Liver Int..

[39] Fumeron F., Péan F., Driss F., Balkau B., Tichet J., Marre M., Grandchamp B., Insulin Resistance Syndrome (DESIR) Study Group (2006). Ferritin and transferrin are both predictive of the onset of hyperglycemia in men and women over 3 years: The data from an epidemiological study on the Insulin Resistance Syndrome (DESIR) study. Diabetes Care.

[40] Ryoo J.H., Park S.K., Jung J.Y. (2015). Elevated Fasting Insulin Level Significantly Increases the Risk of Microalbuminuria. Circ. J..

[41] Loebstein R., Lehotay D.C., Luo X., Bartfay W., Tyler B., Sher G.D. (1998). Diabetic nephropathy in hypertransfused patients with beta-thalassemia. The role of oxidative stress. Diabetes Care.

[42] Vaziri N.D. (2016). Safety Issues in Iron Treatment in CKD. Semin. Nephrol..

[43] Ikeda Y., Enomoto H., Tajima S., Izawa-Ishizawa Y., Kihira Y., Ishizawa K., Tomita S., Tsuchiya K., Tamaki T. (2013). Dietary iron restriction inhibits progression of diabetic nephropathy in db/db mice. Am. J. Physiol.-Renal Physiol..

[44] Rajapurkar M.M., Hegde U., Bhattacharya A., Alam M.G., Shah S.V. (2013). Effect of deferiprone, an oral iron chelator, in diabetic and non-diabetic glomerular disease. Toxicol. Mech. Methods.

[45] Chartampilas E. (2018). Imaging of nonalcoholic fatty liver disease and its clinical utility. Hormones.

[46] Groop P.H., Forsblom C., Thomas M.C. (2005). Mechanisms of disease: Pathway-selective insulin resistance and microvascular complications of diabetes. Nat. Clin. Pract. Endocrinol. Metab..

[47] Rashidbeygi E., Safabakhsh M., Delshad Aghdam S., Mohammed S.H., Alizadeh S. (2019). Metabolic syndrome and its components are related to a higher risk for albuminuria and proteinuria: Evidence from a meta-analysis on 10,603,067 subjects from 57 studies. Diabetes Metab. Syndr..

[48] Singh B., Saxena A. (2010). Surrogate markers of insulin resistance: A review. World J. Diabetes.

[49] Nestler J.E., Barlascini C.O., Tetrault G.A., Fratkin M.J., Clore J.N., Blackard W.G. (1990). Increased transcapillary escape rate of albumin in nondiabetic men in response to hyperinsulinemia. Diabetes.

[50] Redon J., Miralles A., Pascual J.M., Baldó E., Garcia Robles R., Carmena R. (1997). Hyperinsulinemia as a determinant of microalbuminuria in essential hypertension. J. Hypertens..

[51] Gordeuk V.R., Reboussin D.M., McLaren C.E., Barton J.C., Acton R.T., McLaren G.D., Harris E.L., Reiss J.A., Adams P.C., Speechley M. (2008). Serum ferritin concentrations and body iron stores in a multicenter, multiethnic primary-care population. Am. J. Hematol..

[52] Jehn M., Clark J.M., Guallar E. (2004). Serum ferritin and risk of the metabolic syndrome in U.S. adults. Diabetes Care.

[53] Branten A.J., Swinkels D.W., Klasen I.S., Wetzels J.F. (2004). Serum ferritin levels are increased in patients with glomerular diseases and proteinuria. Nephrol. Dial. Transplant..

[54] De Mello V.D., Zelmanovitz T., Perassolo M.S., Azevedo M.J., Gross J.L. (2006). Withdrawal of red meat from the usual diet reduces albuminuria and improves serum fatty acid profile in type 2 diabetes patients with macroalbuminuria. Am. J. Clin. Nutr..

[55] Facchini F.S., Hua N.W., Stoohs R.A. (2002). Effect of iron depletion in carbohydrate-intolerant patients with clinical evidence of nonalcoholic fatty liver disease. Gastroenterology.

[56] Fernández-Real J.M., Peñarroja G., Castro A., García-Bragado F., Hernández-Aguado I., Ricart W. (2002). Blood letting in high-ferritin type 2 diabetes: Effects on insulin sensitivity and beta-cell function. Diabetes.

[57] Yoneda M., Fujii H., Sumida Y., Hyogo H., Itoh Y., Ono M., Eguchi Y., Suzuki Y., Aoki N., Kanemasa K. (2011). Platelet count for predicting fibrosis in nonalcoholic fatty liver disease. J. Gastroenterol..

[58] Milovanovic Alempijevic T., Stojkovic Lalosevic M., Dumic I., Jocic N., Pavlovic Markovic A., Dragasevic S., Jovicic I., Lukic S., Popovic D., Milosavljevic T. (2017). Diagnostic Accuracy of Platelet Count and Platelet Indices in Noninvasive Assessment of Fibrosis in Nonalcoholic Fatty Liver Disease Patients. Can. J. Gastroenterol. Hepatol..

[59] Angulo P., Hui J.M., Marchesini G., Bugianesi E., George J., Farrell G.C., Enders F., Saksena S., Burt A.D., Bida J.P. (2007). The NAFLD fibrosis score: A noninvasive system that identifies liver fibrosis in patients with NAFLD. Hepatology.

[60] Ünübol M., Ayhan M., Güney E. (2012). The relationship between mean platelet volume with microalbuminuria and glycemic control in patients with type II diabetes mellitus. Platelets.

[61] Papanas N., Symeonidis G., Maltezos E., Mavridis G., Karavageli E., Vosnakidis T., Lakasas G. (2004). Mean platelet volume in patients with type 2 diabetes mellitus. Platelets.

[62] Hamilos M., Petousis S., Parthenakis F. (2018). Interaction between platelets and endothelium: From pathophysiology to new therapeutic options. Cardiovasc. Diagn. Ther..

[63] Ceriello A. (1993). Coagulation activation in diabetes mellitus: The role of hyperglycaemia and therapeutic prospects. Diabetologia.

[64] Ferreira D., Severo M., Araújo J., Barros H., Guimarães J.T., Ramos E. (2019). Association between insulin resistance and haematological parameters: A cohort study from adolescence to adulthood. Diabetes Metab. Res. Rev..

[65] Abdel-Moneim A., Mahmoud B., Sultan E.A., Mahmoud R. (2019). Relationship of leukocytes, platelet indices and adipocytokines in metabolic syndrome patients. Diabetes Metab. Syndr..

[66] Zhao F., Yan Z., Meng Z., Li X., Liu M., Ren X., Zhu M., He Q., Zhang Q., Song K. (2018). Relationship between mean platelet volume and metabolic syndrome in Chinese patients. Sci. Rep..

[67] Nelson J.E., Klintworth H., Kowdley K.V. (2012). Iron metabolism in Nonalcoholic Fatty Liver Disease. Curr. Gastroenterol. Rep..

[68] Ko C., Siddaiah N., Berger J., Gish R., Brandhagen D., Sterling R.K., Cotler S.J., Fontana R.J., McCashland T.M., Han S.H. (2007). Prevalence of hepatic iron overload and association with hepatocellular cancer in end-stage liver disease: Results from the National Hemochromatosis Transplant Registry. Liver Int..

[69] Marchesini G., Brizi M., Morselli Labate A.M., Bianchi G., Bugianesi G., McCullough A.J., Forlani G., Melchionda N. (1999). Association of non-alcoholic fatty liver disease to insulin resistance. Am. J. Med..

[70] Marchesini G., Brizi M., Bianchi G., Tomassetti S., Bugianesi E., Lenzi M., McCullough A.J., Natale S., Forlani G., Melchionda N. (2001). Nonalcoholic fatty liver disease: A feature of the metabolic syndrome. Diabetes.

[71] Sanyal A.J., Campbell-Sargent C., Mirshahi F., Rizzo W.B., Contos M.J., Sterling R.K., Luketic V.A., Shiffman M.L., Clore J.N. (2001). Nonalcoholic steatohepatitis: Association of insulin resistance and mitochondrial abnormalities. Gastroenterology.

[72] Yki-Järvinen H. (2010). Liver fat in the pathogenesis of insulin resistance and type 2 diabetes. Dig. Dis..

[73] Fabbrini E., Magkos F., Mohammed B.S., Pietka T., Abumrad N.A., Patterson B.W., Okunade A., Klein S. (2009). Intrahepatic fat, not visceral fat, is linked with metabolic complications of obesity. Proc. Natl. Acad. Sci. USA.

[74] Bugianesi E., Gastaldelli A., Vanni E., Gambino R., Cassader M., Baldi S., Ponti V., Pagano G., Ferrannini E., Rizzetto M. (2005). Insulin resistance in non-diabetic patients with non-alcoholic fatty liver disease: Sites and mechanisms. Diabetologia.

[75] Musso G., Gambino R., Tabibian J.H., Ekstedt M., Kechagias S., Hamaguchi M., Hultcrantz R., Hagström H., Yoon S.K., Charatcharoenwitthaya P. (2014). Association of non-alcoholic fatty liver disease with chronic kidney disease: A systematic review and meta-analysis. PLoS Med..

[76] Slee A.D. (2012). Exploring metabolic dysfunction in chronic kidney disease. Nutr. Metab..

[77] Tripepi G., Jager K.J., Dekker F.W., Zoccali C. (2010). Selection Bias and Information Bias in Clinical Research. Nephron Clin. Pract..

